# A Validated Method for the Determination of Carnosic Acid and Carnosol in the Fresh Foliage of *Salvia rosmarinus* and *Salvia officinalis* from Greece

**DOI:** 10.3390/plants11223106

**Published:** 2022-11-15

**Authors:** Charikleia Paloukopoulou, Anastasia Karioti

**Affiliations:** Laboratory of Pharmacognosy, School of Pharmacy, Aristotle University of Thessaloniki, University Campus, 54124 Thessaloniki, Greece

**Keywords:** Salvia rosmarinus, Rosmarinus officinalis, Salvia officinalis, HPLC-PDA-MS, carnosic acid, carnosol

## Abstract

In the framework of a project aiming at identifying genotypes of Greek rosemary and sage producing high amounts of carnosic acid, an HPLC-PDA method was developed for the determination of the main antioxidant in the fresh leaves. To this end, an effective and repeatable extraction process of the labile diterpene was developed to ensure a good extraction yield. A fast RP-HPLC protocol was developed and optimized to allow for a short and reliable analysis of the unstable target constituent. The HPLC-PDA method was validated for precision and accuracy according to ICH guidelines. Finally, the overall method was validated for precision and accuracy at three concentration levels. The precision was acceptable with % RSD values ranging between 1.42 and 4.35. The recovery ranged between 85.1% and 104.6% with RSD values < 5%, within the acceptable limits. The developed assay was fast and simple and allowed for the fast and accurate determination of carnosic acid and carnosol in the fresh herbs. The methodology was applied to the quantitative analysis of several cultivated samples of *S. rosmarinus* and *S. officinalis,* and some of them were revealed to be promising starting materials for the development of Greek genotypes rich in carnosic acid.

## 1. Introduction

Carnosic acid is an abietan-type diterpene, restricted to specific genera of the Lamiaceae family, especially in rosemary (*Salvia rosmarinus* Spell. *syn Rosmarinus officinalis*) and common salvia (*Salvia officinalis*) [1,2,3]. The presence of a catechol moiety confers high antioxidative properties, while the terpene skeleton renders the molecule soluble in fat. These two characteristics, along with antimicrobial properties, make carnosic acid and its derivatives excellent antioxidants. Indeed, they extend the shelf life of lipid-rich products, and their performance is superior when compared to synthetic antioxidants [4]. These activities are widely recognized, and numerous patents with applications of rosemary extracts enriched in carnosol and carnosic acid have been published [2]. In 2008, the European Food Safety Authority (EFSA) authorised extracts of Rosemary (E 392) as food additives [5], with margins of safety ranging from 100–2000 and 200–3000 mg (carnosic acid plus carnosol) for children and adults, respectively [6]. This authorization paved the way for the exploitation of carnosic acid and its derivatives in the food industry, as preservatives. Aside from the protective role it has on foodstuff, there is growing evidence of its beneficial role in health, especially as an anti-inflammatory agent. Carnosic acid has been shown to decrease NO and TNF-α production and inflammatory cytokines, in several in vitro and in vivo models, to downregulate COX2 expression, and to lower the transcriptional level of inflammatory genes [7,8,9]. Due to its antioxidant and anti-inflammatory properties, it is intensively studied in other conditions where oxidative stress plays an important role, such as cancer, neurodegenerative disorders [10,11,12,13]. Its beneficial role in health seems to be supported by few bioavailability data whereby, carnosic acid is transported through Caco-2 cells and is present in the digestive tract, especially in the cecum and colon. Trace quantities of carnosic acid-derived metabolites were reported also in the brain [14,15]. These effects seem to be potentiated in presence of other constituents in the extracts [16]. This could be due to additive or synergistic effects, or to the concomitant presence of many antioxidant principles which protects each other from degradation.

In view of the wide applications of carnosic acid and its enriched Rosemary/Sage extracts in the pharmaceutical, food and cosmetics sectors, improved genotypes of *S. rosmarinus* and *S. officinalis* with high content in carnosic acid are desirable for further exploitation in the industry. In the framework of a research program aiming to identify the Greek genotypes of rosemary and sage with a high yield in carnosic acid, an HPLC-PDA-MS analytical protocol for the determination of carnosic acid and its biosynthetic product, carnosol, in the fresh foliage tissues was developed and optimized. A thorough literature research demonstrated the lack of analytical protocols in determining the amounts of these antioxidants in fresh plant material, which is essential in a metabolomic analytical approach. Indeed, there are only a few reports on the qualitative and quantitative profile of the fresh but lack method validation. Oliveira et al. [17] developed a UHPLC-DAD multi-method for the determination of phenolics in aromatic plants, among them carnosic acid and carnosol, but both the HPLC and the extraction are general for a vast variety of phenolic compounds and not specific for these antioxidant diterpenes. Similarly, Choi et al. [18] developed an HPLC-PDA method for the determination of the antioxidant principles of rosemary in food matrices and the extraction protocol was optimized for the separation of these compounds from fat-containing matrices such as oils, meat products, and salad dressings.

The present study aimed to fill the above-mentioned gap by developing an analytical methodology, suitable for metabolomic studies (i.e., a large number of samples, over 500) for the determination primarily of carnosic acid and secondarily of carnosol in the fresh plant material of *S. rosmarinus* and *S. officinalis*. In this framework, it was important to set up a fast and repeatable extraction protocol and a short HPLC method taking into account the labile nature of both compounds, the big number of analyses, and to validate it according to the ICH (International Council for Harmonisation) guidelines.

## 2. Results

**Figure 1:**
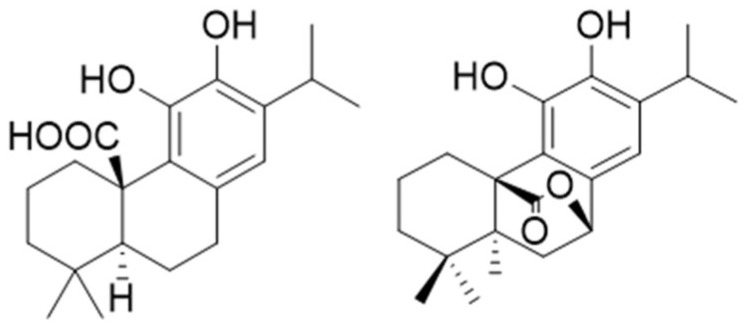
Structures of Carnosic acid and Carnosol.

A suitable extraction protocol was developed, and an RP HPLC method was set up and validated. Τhe whole methodology, including the extraction process, was validated according to ICH guidelines [19,20]. The assay took into consideration carnosic acid which is the biosynthetic precursor of carnosol (Figure 1) and of other antioxidant diterpenes [21].

Preliminary tests with vortex-assisted extraction and sonication-assisted extraction showed that the highest yield was achieved with sonication for both fresh plants. Furthermore, a series of solvents were tested including aqueous methanol 75%, 100% methanol, 100% ethanol, and 100% acetone (Table S1, Supplementary Data). The best extraction yield in terms of repeatability and effectiveness was observed for 100% acetone, so this solvent was used in the final analytical protocol (Table S1, Supplementary Data). Finally, an experiment using different extraction durations revealed that the best extraction yield is achieved after 15 min but less than 20 (Figure S1, Supplementary Data). Therefore, a 15 min sonication with 100% acetone was the final extraction protocol.

The developed HPLC method was also validated to ensure its suitability for the analyses. It showed good selectivity, peak shape, and symmetry, while it had good linearity with *r*^2^ values for the two standards ranging from 0.9992 to 0.9997 (Table 1).

The intra-day variations were calculated by measuring the amounts of the two representative diterpenes at three different concentration levels (50%, 100%, and 200% of the real sample) at six replicates (Table 2). For each concentration level, mean, standard deviation (SD) and relative standard deviation (RSD) were calculated. RSD values ranged between 0.20% and 1.36%, which is within acceptable limits. Due to the degradation of the diterpenes in 24 h, the inter-day variation (i.e., in four consecutive days) was not considered.

The accuracy of the HPLC method was also acceptable, with recoveries ranging from 94.5–105.8% and RSD between 0.46 and 4.74% (Table S2). The overall method (extraction step included) was tested for repeatability (in six independent replicates) and for accuracy at three different concentration levels of the main analytes. The experiment was carried out with a sample of *S. rosmarinus*. Repeatability with six independent replicates was also tested with an abundant sample of *S. officinalis*. The analytical protocol showed good repeatability with small RSD (values ranging between 1.42 and 4.35% for *S. rosmarinus* and between 2.54 and 3.34% for *S. officinalis*) (Table 2).

To assess the accuracy of the overall methodology, a recovery experiment was carried out at three different concentration levels. The values obtained ranged between 95.75% and 104.6% (with RSD values < 4.74%) (Table S3) which are within the acceptable limits for matrix spike recovery, especially if the unstable nature of the antioxidant diterpenes is considered.

Once the analytical protocol was established, the quantitation of fresh samples of *S. rosmarinus* and *S. officinalis* was possible. Twenty samples of cultivated *S. rosmarinus* and twenty samples of *S. officinalis* were analyzed for their content in carnosic acid and carnosol. Quantitative results are shown in Table 3 and a representative chromatogram is presented in Figure S2 (Supplementary Data).

## 3. Discussion

Sampling for the preliminary studies and for the setup of the analytical method was done according to good practices in metabolomic studies [22]. Parameters that were taken into consideration were the developmental stage of the plants the quantity of the foliage (at least 5 g) and also the harvesting method which included the deactivation of the enzymes by use of liquid nitrogen and dry ice. HPLC-PDA analyses of both first-year cultivated *S. rosmarinus* and *S. officinalis* leave extracts showed the predominance of two main constituents, carnosic acid and carnosol. By applying the normalization method at 280 nm, the content of these two diterpenes was estimated to be approximately 80% of total diterpenes, with carnosic acid being the main compound with a ratio over carnosol at least 2:1. Therefore, the developed assay (extraction and HPLC) was based mainly on carnosic acid. Due to the chemical instability of carnosic acid and carnosol under light, high temperatures and most protic solvents, an optimization of the extraction process was necessary. Parameters which were taken into consideration were the solvent, the time, and method of the extraction, the overall time of analysis. Our main goal was to ensure fast, effective and repeatable extraction of the main analytes and at the same time to ensure their chemical stability. Initially, a comparison between vortex-assisted extraction and sonication-assisted extraction was performed. The highest yield was observed for sonication for both fresh plants. Bath ultrasound was also more practical, since it permitted the extraction of several samples at the same time, which is desirable considering the high number of samples and the repetitions required in metabolomic analysis. To avoid degradation of the target compounds an ultrasound bath at 35 KHz and not 40 KHz was applied, as previously suggested [23], while the water temperature was maintained below 50 °C. To optimize the extraction a series of solvents were tested including aqueous methanol 75%, 100% methanol, 96% ethanol, and acetone 100% (Table S1, Supplementary Data). All extractions were performed in triplicate to assess the effectiveness and repeatability of the extraction for each solvent. Methanol 75% was chosen in order to compare with previous methodologies applied in *S. officinalis* metabolomic analyses [24]. However, this solvent mixture had the lowest extraction yield, especially for carnosic acid, as shown by our HPLC-PDA analysis. Furthermore, both carnosol and carnosic acid are reported to be sensible to protic solvents, especially water, methanol, and ethanol, whereas in methanol transformation of carnosic acid to carnosol occurs [25,26,27,28]. For this reason, the use of water as an extraction solvent was not considered further. HPLC-PDA results demonstrated that methanol 100% is better for the extraction of carnosol, while acetone is more suitable for the extraction of carnosic acid. In contrast, ethanol 96% was not effective and at the same time had a bad repeatability for the extraction of the metabolites (RSD values of 16.9 and 18.1, Table S1, Supplementary Data). Paniwyk et al. [23] proposed that methanol is a better extraction agent than denaturated ethanol. When the same group applied a combination of ultrasound and mechanical stirring, the extraction was improved and under such conditions denaturated ethanol gave better results, especially for carnosic acid. Indeed, Mulinacci et al. [29] applied, with success, a combination of both techniques for the overall evaluation of antioxidants in *S. rosmarinus* also taking into consideration the low toxicity of ethanol. In our case, we had to compromise and consider a large number of samples (over 360 for both plant species), so the necessity for a fast extraction procedure excluded further steps with additional techniques or solvents. The difference between ethanol and methanol might be attributed to the different viscosity of the solvents. In line with this, acetone 100%, being the less viscous solvent, achieves better penetration of the tissues leading to a fast and effective extraction. Furthermore, having a lower polarity than methanol/ethanol, it is better for the extraction of the medium polarity carnosic acid, while being an aprotic solvent ensures better stability of both the analytes. Another point to consider was the fact that in a metabolomic study analytical variations should always be minimized compared to the biological ones, in order to get credible results. Since acetone 100% gave the best results in terms of repeatability, this solvent was used in the final analytical protocol. In the last step, the time of sonication was considered. Results showed that the extracted amounts of carnosic acid were increased up to 15 min, after which a decrease was observed, which was more intense after 30 min of extraction (Figure S2, Supplementary Data). This is probably due to the thermal degradation of the labile compound. Therefore, 15 min was selected as the maximum time, while a second extraction cycle was excluded, to avoid further degradation of the labile metabolites and keep the time of analysis to a minimum. In summary, ultrasonic extraction of the samples in 100% acetone for 15 min gave the best extraction efficiency and this was the final analytical protocol.

Due to the well-known instability of both the analytes (carnosic acid and carnosol) in solvents, the HPLC method had to be fast and effective in order to perform batch analyses in a relatively short time. Several experiments with the same sample injected over different times showed a small but observable degradation of the diterpenes in 24 h. For this reason, the inter-day variation (i.e., the consideration of the same sample in four consecutive days) was not considered. In contrast, several experiments with the same sample over different hours during the same day (intra-day variation) showed that analyses can be executed with safety in a time margin of 5–10 h. Indeed, the results of the intra-day variation demonstrated that the method gives credible results, especially when considering that there is only one extraction step and that the compounds tend to degrade in solution. This is also reflected in the accuracy experiment which was also within the acceptable limits for matrix spike recovery. Therefore, for a credible analysis of the samples, a 5 h duration of a batch of analyses corresponding to ten samples is proposed. Furthermore, the overall method, comprising the extraction step was tested in six independent replicates to ensure adequate extraction of the target analytes. This test was performed in both plant materials (*S. rosmarinus* and *S*. *officinalis*) and according to the results, the methodology proved effective as it provided a repeatable result.

As the studied plants were plants of the first year, the extracts contained mainly carnosic acid and carnosol, while rosmarinic acid, the important antioxidant phenolic was in traces. The studied samples varied between them, as expected due to genetic or environmental variability. To the best of our knowledge, this is the first report on the content of Greek *S. rosmarinus* in carnosic acid/carnosol. Previous reports on *S. rosmarinus* from Greece [30,31,32,33], concern the phenolic profile or the total phenolic content. Likewise, for *S. officinalis*, there are few reports that focus mainly on the extracts or essential oils, rather than the chemical content of the plant. Sarrou et al. [34] were the first to perform an analysis of seven *S. officinalis* populations; however, the extraction method was adapted to the bulk of phenolic metabolites, employing 80% methanol which was ineffective for the extraction of the less polar carnosic acid. From these preliminary quantitative results, it is demonstrated that both plants are a good source of carnosic acid and are promising starting materials for the development of Greek genotypes rich in carnosic acid.

## 4. Materials and Methods

### 4.1. Chemicals

Solvents used for the extraction of diterpenes were of reagent grade, whereas the solvents used for HPLC analysis were of HPLC grade. All solvents were purchased from Sigma-Aldrich. Water was purified by a Milli-Qplus system from Millipore. Carnosic acid and carnosol dihydrate (purity 90%) were purchased from Extrasynthèse.

### 4.2. Optimization of Extraction and HPLC

In order to compare vortex with ultrasonic extraction, 500 mg of fresh plant material (*S. rosmarinus*/*S. officinalis*) of the same origin were vortexed/sonicated for 10 min in 10 mL of 100% methanol. The extracts were collected, filtered, adjusted to 10 mL in a volumetric flask, and subjected to HPLC-PDA-MS. For the selection of the best extraction solvent, the same experiment was carried out using the same plant material (500 mg) which was extracted in parallel with aqueous methanol 75%, 100% methanol, 100% ethanol, and 100% acetone. Finally, the duration of the extraction was tested: the same amounts (500 mg) of the same sample were sonicated in parallel in 10mL of acetone 100% for 5, 10, 15, 20, and 30 min. The extracts were collected, adjusted to 10mL in a volumetric flask and subjected to HPLC-PDA-MS.

### 4.3. Plant Samples and Sample Preparation for HPLC Analysis

For the development of the analytical method, five samples of *S. rosmarinus*leaves were harvested at the end of June 2019 from an outdoor experimental field collection, located in the region of Piperia Aridea (latitude 40.964263° N, longitude 22.017363° E). These were pooled in order to obtain a homogenous sample of an adequate quantity to carry out the development and the validation of the methodology.

For the quantitative analyses, twenty samples of cultivated *S. rosmarinus* leaves (RO1-RO20) were harvested in the end of August 2019 from the same experimental field, while the samples of cultivated *S. officinalis* leaves (SO1-SO20) were harvested at August 2020. To inhibit enzymatic activity, the leaves were immediately immersed in situ (in the field), in liquid nitrogen and transported with dry ice to the laboratory and stored at −80 °C before further treatment. The samples were prepared according to the final analytical protocol: more than 10 g of each fresh plant sample was ground with a mortar and pestle with liquid N_2_ and mixed to obtain homogenous samples. Approximately 500 mg of the fresh and grounded *S. rosmarinus* or *S. officinalis* leaves were ultrasonicated with 10 mL of 100% acetone for 15 min once. The samples were filtered through a paper filter and the filtrates were adjusted to 50.0 mL using 100% acetone. The solutions were filtered through Nylon filters (0.45 µm pore size) and immediately injected.

### 4.4. HPLC Analysis

#### 4.4.1. HPLC-PDA-MS Analysis Instrumentation

The analysis was carried out on an LC-PDA-MS Thermo Finnigan system (LC Pump Plus, Autosampler, Surveyor PDA Plus Detector) interfaced with an ESI MSQ Plus (Thermo Finnigan, MA, USA) and equipped with an Xcalibur software. The mass spectrometer was operated in both negative and positive ionization modes in the range from *m*/*z* 100 to 1000. Gas temperature was at 350 °C, the nitrogen flow rate at 10 L/min, and capillary voltage 3000 V. The cone voltage was at 75 V. A SB-Aq RP-C18 column (5 µm, 150 mm × 3 mm, Agilent Agilent Technologies, Palo Alto, CA, USA) maintained at 30 °C was used for separation. The mobile phase consisted of H_2_O containing 0.05% formic acid (pH 2.8–3.0) (A) and acetonitrile (B) at a flow rate of 0.4 mL/min. Samples were analyzed with the following gradient: 0–10 min, 50% A; 10–20 min, to 42% A; 20–25 min 50% A. A volume of 5 μL was injected. The UV–vis spectra were recorded between 220 and 600 nm and the chromatographic traces were registered at 280 nm. Due to the presence of lipophilic compounds in the samples, the column was flushed with 100% acetonitrile for thirty minutes every five analyses.

#### 4.4.2. Identification of Peaks and Peak Purity—Quantitative Determination of Diterpenoids

Identification of the main constituents was performed by a combination of HPLC-PDA-MS and reference standards. Peak purity was checked by examination of the UV and MS spectra. For the quantitative determination of carnosic acid and carnosol, the external standard method was applied using carnosic acid and carnosol. Carnosic acid and carnosol were accurately weighed (1.9 mg and 2.2 mg, respectively) and dissolved in 10 mL of DMSO to give stock solutions which were kept at −80 °C. A series of dilutions were prepared in acetone immediately before analysis. New stock solutions were prepared every 4 days. The regression curve was obtained by measuring each point in triplicate. Measurements were performed at 280 nm which is the mean maximum absorbance of these compounds. Quantitative results (Table 3) are expressed as μg/g of fresh weight of leaves.

#### 4.4.3. Method Validation

##### Linearity, LOD, LOQ, Precision, and Accuracy of the HPLC Method

The linearity range of responses of the standards carnosic acid and carnosol was determined at seven concentration levels with three injections for each level. For carnosic acid, calibration graphs were recorded with amounts ranging from 18.6 × 10^−3^ mg to 0.37 mg, while for carnosol from 8.8 ng to 22 ng. Solutions of the standards (LOQ included) were prepared at different concentrations ranging from 3.4 × 10^−3^ mg/mL to 8.5 × 10^−3^ mg/mL and from 0.9 × 10^−3^ mg/mL to 2.2 × 10^−3^ mg/mL for carnosic acid and carnosol, respectively, and were injected into HPLC (injection volumes varying from 2 to 8 μL). The limit of detection (LOD) and quantification (LOQ) under the chromatographic conditions were determined by injecting a series of standard solutions until the signal-to-noise (S/N) ratio was 3 for LOD and 10 for LOQ. To evaluate the repeatability of the HPLC method on real samples, the extract of the more abundant sample was used and was analysed at three different concentration levels corresponding to 200%, 100%, and 50% in the range of the calibration curve. To assess intra-day variability, the sample was analysed in six replicates within one day. Inter-day variability was not examined due to the instability of the standards. The contents of the two antioxidants were measured to calculate the relative standard deviation (RSD). To test the accuracy of the HPLC method a known amount of a freshly prepared mix of standard solutions of carnosic acid and carnosol was added into a certain amount of sample at three different concentration levels in the range of the calibration curve. The samples were measured in triplicates, and the amount of the standards added was calculated by subtraction (in total, 12 injections).

##### Precision and Accuracy of the Overall Method (Including Extraction Procedure)

To check the repeatability of the method at three different concentration levels, samples of 750, 500, and 250 mg of fresh leaves were used. Six different extracts for each concentration level were prepared according to the protocol described above and injected twice into the HPLC. The mean, standard deviation, and relative standard deviation (RSD) were calculated. Results were expressed as RSD. The accuracy of the overall method was determined by a recovery experiment. Carnosic acid (26.29 mg) and carnosol (15.0 mg) were mixed in a volumetric flask of 20 mL to obtain a concentrated stock solution. Different amounts of this stock solution (2.0, 1.0 and 0.5 mL) were added to 70% of the fresh leaves of *S. rosmarinus* (350 mg) to achieve three different concentration levels, corresponding approximately 90%, 110%, and 150% of the two analytes. The samples were treated according to the final method. Extractions were repeated three times for each concentration level.

## 5. Conclusions

An analytical protocol, including a suitable extraction and an HPLC-UV method, was developed to determine the content of carnosic acid in the fresh leaves of rosemary and sage from Greece. The developed extraction protocol based on ultrasonication and acetone 100% could guarantee the chemical stability of the target diterpenoids and ensured, at the same time, good extraction yield and repeatability. Both the HPLC and the whole method were validated according to the ICH guidelines. The accuracy was acceptable (recovery < 5%, and RSD < 5%, less than 15% for values over LOQ), and along with the overall precision of the methodology (RSD < 5%), confirms the efficiency of the method. This method is a suitable analytical tool for the determination of carnosic acid in a large number of plant tissues of both *S. rosmarinus* and *Salvia officinalis.* Preliminary quantitative studies showed that both plants are a good source of carnosic acid and are promising starting materials for the development of Greek genotypes rich in carnosic acid.

## Figures and Tables

**Table 1:** Calibration equations, LODs, LOQs, and repeatability calibration curves for compounds carnosic acid and carnosol.

	Calibration Equation	Correlation Coefficient	LOD (ng)	LOQ (ng)	RSD% (*n* = 3)
Carnosic Acid	y = 2 × 10^−7^x − 0.0013	1.0	0.68	2.0	1.43
Carnosol	y = 2 × 10^−7^x + 0.0027	0.999	1.8	4.4	4.16

**Table 2:** Precision data of the HPLC method of the overall method (extraction included) expressed as μg/g ± SD (RSD).

Compound	Precision HPLC, Intra-Day (*n* = 6)		Precision, Overall (*n = 6*)		
	Concentration Level	μg/g ± SD (% RSD)	Amount of Fresh Plant	*S. rosmarinus* μg/g ± SD (% RSD)	*S. officinalis* μg/g ± SD (% RSD)
Carnosic Acid	200%	9626.59 ± 50.51 (0.52)	200%	9762.19 ± 237.82 (2.44)	4709.06 ± 137.74 (2.92)
	100%	9786.07 ± 87.35 (0.89)	100%	9690.86 ± 272.21 (2.81)	4627.68 ± 147.85 (3.19)
	50%	9529.74 ± 50.62 (0.53)	50%	9858.55 ± 167.04 (1.69)	4544.19 ± 151.84 (3.34)
Carnosol	200%	4135.29 ± 56.18 (1.36)	200%	4250.55 ± 60.44 (1.42)	636.27 ± 19.88 (3.12)
	100%	4319.67 ± 100.89 (2.34)	100%	4277.96 ± 186.14 (4.35)	601.67 ± 15.31 (2.54)
	50%	4113.35 ± 21.74 (0.53)	50%	4176.88 ± 78.96 (1.89)	615.71 ± 15.90 (2.58)

**Table 3:** Amounts of carnosic acid and carnosol expressed as μg/g (fresh weight) in the samples of *S. rosmarinus* and *S. officinalis* leaves (*n* = 3).

	*S. rosmarinus*			*S.* *officinalis*	
	Carnosic Acid	Carnosol		Carnosic Acid	Carnosol
Plant Sample	Mean Value ± SD (RSD)		Plant Sample	Mean Value ± SD (RSD)	
RO1	9786.1 ± 87.30 (0.89)	4319.7 ± 100.90 (2.34)	SO1	3676.60 ± 21.38 (0.58)	472.04 ± 3.90 (0.83)
RO2	12,173.01 ± 52.48 (0.43)	4470.37 ± 15.35 (0.34)	SO2	4367.27 ± 23.92 (0.55)	649.41 ± 2.66 (0.41)
RO3	10,827.56 ± 907.90 (8.38)	3545.94 ± 138.10 (3.89)	SO3	5458.12 ± 21.77 (0.40)	866.19 ± 0.13 (0.01)
RO4	9413.33 ± 30.60 (0.32)	3559.66 ± 188.04 (5.28)	SO4	4681.52 ± 25.87 (0.55)	600.55 ± 0.90 (0.15)
RO5	7472.86 ± 102.70 (1.37)	2145.52 ± 20.58 (0.96)	SO5	3001.75 ± 115.98 (3.86)	435.30 ± 1.30 (0.30)
RO6	8833.70 ± 9.68 (0.11)	3629.86 ± 40.07 (1.11)	SO6	3676.36 ± 10.19 (0.28)	640.39 ± 1.19 (0.19)
RO7	10,045.85 ± 105.36 (1.05)	3319.29 ± 85.53 (2.58)	SO7	3662.32 ± 28.47 (0.78)	633.50 ± 1.40 (0.22)
RO8	9642.48 ± 38.26 (0.40)	3534.29 ± 29.66 (0.84)	SO8	5833.98 ± 73.08 (1.25)	981.28 ± 2.02 (0.20)
RO9	7514.85 ± 108.07 (1.44)	3143.29 ± 25.97 (0.83)	SO9	3790.78 ± 28.88 (0.76)	809.93 ± 18.73 (2.31)
RO10	5827.51 ± 2.38 (0.04)	2716.66 ± 5.59 (0.21)	SO10	5452.92 ± 172.88 (3.17)	1085.41 ± 1.16 (0.11)
RO11	7576.98 ± 53.51 (0.70)	3137.02 ± 16.27 (0.52)	SO11	4476.82 ± 29.72 (0.66)	1044.45 ± 0.65 (0.06)
RO12	6201.98 ± 25.98 (0.42)	2741.18 ± 9.22 (0.34)	SO12	7033.23 ± 52.52 (0.75)	880.33 ± 20.45 (2.32)
RO13	8089.95 ± 63.86 (0.79)	2751.88 ± 96.15 (3.49)	SO13	4398.15 ± 15.29 (0.35)	831.13 ± 12.30 (1.48)
RO14	10,625.26 ± 181.58 (1.71)	3680.84 ± 9.71 (0.26)	SO14	6295.81 ± 26.70 (0.42)	794.53 ± 5.53 (0.70)
RO15	5638.22 ± 18.87 (0.33)	3381.14 ± 0.21 (0.01)	SO15	3600.46 ± 20.87 (0.58)	600.11 ± 0.98 (0.160
RO16	6964.89 ±28.36 (0.41)	1392.72 ± 26.99 (1.94)	SO16	5841.15 ± 43.17 (0.74)	879.71 ± 13.57 (1.54)
RO17	6502.17 ± 51.16 (0.79)	2458.34 ± 19.12 (0.78)	SO17	5182.14 ± 28.49 (0.55)	969.52 ± 4.02 (0.42)
RO18	9014.92 ± 9.29 (0.10)	3256.18 ± 1.33 (0.04)	SO18	4753.01 ± 19.29 (0.41)	1077.82 ± 24.77 (2.30)
RO19	6043.98 ± 101.57 (1.68)	1926.25 ± 21.53 (1.12)	SO19	5198.41 ± 20.78 (0.40)	852.76 ± 14.76 (1.73)
RO20	7077.33 ± 120.15 (1.70)	1044.52 ± 13.52 (1.29)	SO20	3178.31 ± 21.99 (0.69)	636.98 ± 5.02 (0.79)

## Data Availability

Not Applicable.

## Supplementary Materials

The following supporting information can be downloaded at: https://www.mdpi.com/article/10.3390/plants11223106/s1. Table S1. Extraction yield of carnosic acid and carnosol expressed as μg/g (fresh weight) in the samples of *S. rosmarinus* in different solvent in three individual extractions of the same sample; Table S2. Accuracy data of the HPLC method at three concentration levels based on carnosic acid and carnosol; Table S3. Accuracy data of the overall method based on carnosic acid and carnosol; Figure S1. Optimization of extraction time; Figure S2. Representative chromatogram of rosemary and sage leave extracts, carnosic acid and carnosol at 280 nm.
