# Supplementary material for: A Validated Method for the Determination of Carnosic Acid and Carnosol in the Fresh Foliage of Salvia rosmarinus and Salvia officinalis from Greece
Source: Plants (Basel). 2022 Nov 15;11(22):3106. doi: 10.3390/plants11223106 (PMC9697906; doi:10.3390/plants11223106)
Supplement: Supplementary file 1 [file plants-11-03106-s001.zip › plants-1978306-supplementary.pdf]

SUPPLEMENTARY DATA

A Validated Method for the Determination of Carnosic Acid and Carnosol in the Fresh Foliage of *Salvia rosmarinus* and *Salvia officinalis* from Greece

Charikleia Paloukopoulou and Anastasia Karioti *

Laboratory of Pharmacognosy, School of Pharmacy, Aristotle University of Thessaloniki, University Campus, 54124 Thessaloniki, Greece

Table S1. Extraction yield of carnosic acid and carnosol expressed as μg/g (fresh weight) in the samples of *S. rosmarinus* in different solvent in three individual extractions of the same sample.

Extraction solvent	Carnosic acid (μg/g FWT) (%RSD)	Carnosol (μg/g FWT) (%RSD)
**Methanol**	7314.21 ± 445.03 (6.08)	4345.93 ± 431.29 (9.92)
**Ethanol 96%**	6531.21 ± 1102.41 (16.88)	2725.35 ± 492.59 (18.07)
**Acetone**	7913.33 ± 275.79 (3.49)	2843.31 ± 3.33 (1.17)

Table S2. Accuracy data of the HPLC method at three concentration levels based on carnosic acid and carnosol.

Compound	Found (μg)	Added (μg)	Recovery (%)	RSD (%, *n* = 3)
**carnosic acid**				
High	0.389	0.410	94.5	1.82
Medium	0.209	0.205	101.8	0.46
Low	0.108	0.103	105.3	3.28
**carnosol**				
High	0.068	0.064	105.8	0.49
Medium	0.031	0.032	97.0	1.88
Low	0.015	0.016	98.9	4.74

Table S3. Accuracy data of the overall method based on carnosic acid and carnosol.

Compound	Found (μg)	Added (μg)	Recovery (%)	RSD (%, *n*=3)
**carnosic acid**				
High	0.26694	0.26292	101.5	4.74
Medium	0.12587	0.13145	95.8	2.61
Low	0.05591	0.06573	85.1	3.69
**carnosol**				
High	0.156	0.150	103.9	3.65
Medium	0.0752	0.0750	100.3	4.20
Low	0.0392	0.0375	104.6	0.62

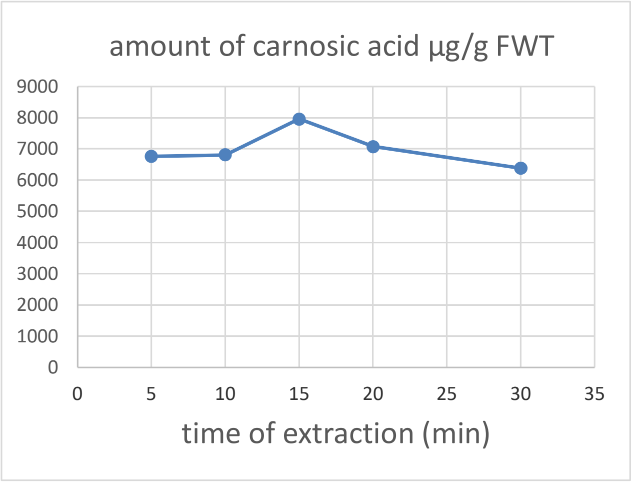

Figure S1. Optimization of extraction time.

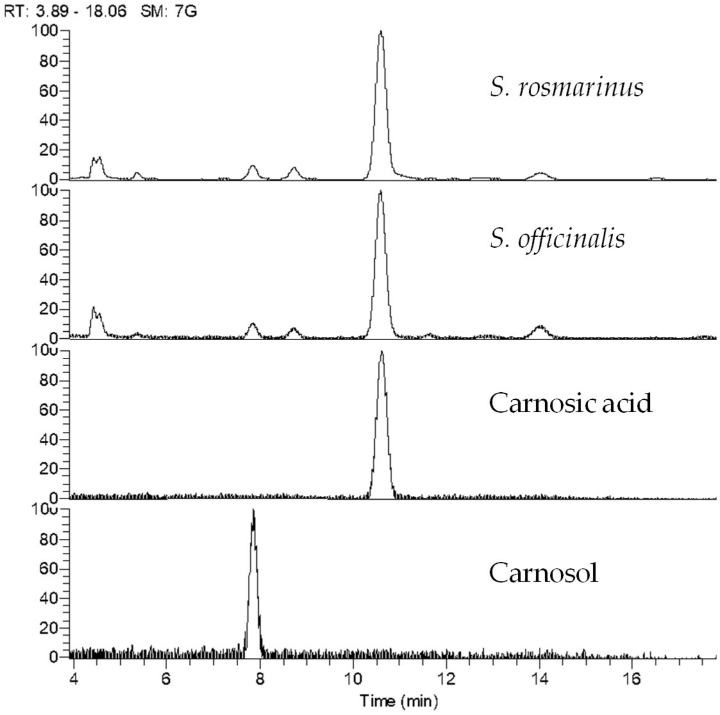

Figure S2. Representative chromatogram of rosemary and sage leave extracts, carnosic acid and carnosol at 280 nm.
